# A Case of Bilateral Choroidal Effusion after XEN Gel Stent Implantation

**DOI:** 10.3390/gels9040276

**Published:** 2023-03-27

**Authors:** Paola Cassottana, Chiara Toma, Cristina Maltese, Viviana Villa, Roberta Ricciarelli, Carlo Enrico Traverso, Michele Iester

**Affiliations:** 1 IRCCS Ospedale Policlinico San Martino, 16132 Genoa, Italy; 2 Clinica Oculistica, Department of Neurosciences, Rehabilitation, Ophthalmology, Genetics, Maternal and Child Health (Dinogmi), University of Genoa, 16132 Genoa, Italy; 3 Department of Experimental Medicine, University of Genoa, 16132 Genoa, Italy

**Keywords:** XEN45 gel stent, serous choroidal detachment, minimally invasive glaucoma surgery, glaucoma, retinal neurodegenerative disease

## Abstract

Purpose: This study aimed to describe a rare case of bilateral choroidal effusion following a XEN45 implantation. Case Report: An 84-year-old man with primary open-angle glaucoma underwent uneventful ab interno XEN45 device implantation in the right eye. The immediate postoperative period was complicated by hypotony and serous choroidal detachment, which were treated and resolved using steroids and cycloplegic drops. Eight months later, the fellow eye underwent the same surgery, which was followed by choroidal detachment that required transscleral surgical drainage. Conclusions: This case highlights the importance of a careful postoperative follow-up and a timely intervention in the context of XEN45 implantation, and suggests that choroidal effusion in one eye may be a risk factor for choroidal effusion in the other eye when undergoing the same type of surgery.

## 1. Introduction

Glaucoma is a chronic progressive neurodegenerative optic neuropathy characterized by the loss of retinal ganglion cells with secondary morphological changes in the optic nerve head and retinal nerve fiber layer. The loss of ganglion cells is strictly related to visual field defects. 

Lowering intraocular pressure (IOP) is the only therapeutic approach known to slow the progression of glaucoma [1], and achieving a lower IOP is the rationale for glaucoma treatment. Based on clinical data such as visual field, optic nerve head appearance, and IOP, an ideal IOP can theoretically be established for each patient. The target IOP will be higher if the patient is elderly or with early/moderate impairment, and it will be lower if the patient is younger or with a moderate/advanced damage. IOP can be reduced with medical treatments, lasers, or surgery. Topical medical treatment is mainly based on four different types of drugs: prostaglandins that increase uveal scleral outflow, beta-blockers and topical carbonic anhydrase inhibitors that decrease aqueous humor production, and alfa2 agonists that decrease the production of aqueous humor and increase trabecular outflow. When the ideal IOP is not achieved with medical treatment, two solutions remain: laser treatment, which reduces the IOP by about 20% and the effect is visible after one month, or surgery. 

Among the different types of glaucoma surgery available, trabeculectomy is the oldest but still the most widely used filtering surgery, although minimally invasive glaucoma surgery (MIGS), which is less effective in reducing IOP and is non-filtering, is preferable in younger patients. 

Glaucoma filtering surgery is one of the most effective strategies for controlling and reducing IOP [2], but several postoperative complications have been reported [3,4]. Identifying changes in anterior chamber depth, IOP levels, and bleb morphology is critical for differentiating early postoperative complications. 

Serous choroidal detachment (CD), also known as choroidal effusion, is a potential complication of glaucoma surgery [5] and usually occurs in the setting of significant postoperative hypotony [6]. Serous fluid from the choriocapillaris accumulates in the virtual space between the choroid and sclera (suprachoroidal space), possibly because of the hydrostatic pressure difference between the hypotonous eye and choroidal vessels [7]. CDs most frequently develop 2–5 days after glaucoma-filtering surgery. While small and peripheral effusions are often asymptomatic, with unchanged visual acuity and little or no anterior chamber shallowing (and are sometimes undiagnosed), large CDs can cause blurred vision, peripheral vision constriction, occasional headaches, and/or nausea. Upon examination, IOP is usually low, and the anterior chamber is shallow due to an anterior shift of the lens–iris diaphragm. Fundus analysis allows for a direct visualization of CDs, which are often peripherally located, with a multilobed appearance and morphology resulting from a strong attachment of the choroid to the vortex vein sites. Fluid-filled lobes of CDs transilluminate (Hagen’s sign) and can be distinguished from hemorrhagic detachments, which appear echo-dense on B-scan ultrasonography [8]. 

Most effusions are self-limiting as the preoperative effects of glaucoma medications decrease, early wound healing occurs, and bleb formation begins. The initial medical treatment of persistent choroidal effusion includes addressing the underlying causes of hypotony, applying cycloplegic eye drops to deepen the anterior chamber by rotating the lens–iris diaphragm, topical corticosteroids, and avoiding physical activities that elevate intrathoracic pressure. In severe cases that are refractory to topical medications, oral glucocorticoids (prednisone 1 mg, gradually tapered until resolution) may be used. If the anterior chamber is extremely shallow or flat, intracameral air and/or cohesive or ultra-cohesive viscoelastic material may be injected via paracentesis to reform the anterior chamber.

Indications for the surgical drainage of CDs include: (i) a flat anterior chamber with persistent lens–corneal contact, (ii) appositional CD (*kissing choroidals*), (iii) combined serous retinal and choroidal detachment, (iv) persistent bleb leak with hypotony, and (v) persistent CD. Effusions can be drained through full-thickness scleral incisions (3.5–4.5 mm posterior to the limbus) parallel or radial to the limbus. The anterior chamber can be filled with fluid or viscoelastic solutions during effusion drainage to promote a more complete drainage, reform the anterior chamber, and elevate the bleb.

With the aim of reducing the complications of glaucoma surgeries and lowering IOP in a safer and less traumatic manner, new devices have been developed, and drainage procedures previously reserved for complex cases of glaucoma or after failed trabeculectomies are now often the primary surgical choice [9]. The XEN45 gel stent, for example, is a 6 mm hydrophilic tube housed in a disposable inserter specifically designed for an ab-interno surgical approach, shunting aqueous humor from the anterior chamber to a filtering bleb in the subconjunctival space [10]. Its small inner lumen (~45 μm) should theoretically avoid early postoperative hypotony [11]; however, post-trabeculectomy complications can also occur after shunting procedures, with the incidence of CD after XEN45 gel stent implantation ranging from 0 to 15% [12,13]. 

Herein, we report a case of bilateral delayed choroidal effusion in a patient who underwent XEN45 implantation. A question arises of the demographic and ocular characteristics associated with the occurrence of CD in patients implanted with the XEN45 gel stent.

## 2. Case Report

**Figure 1:**
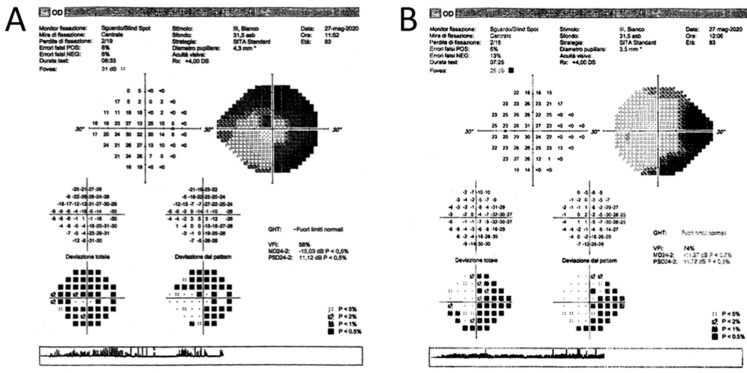
Right (**A**) and left (**B**) eye visual field before XEN45 implantation performed on May 2020. In the right eye, there was a depression of sensitivity with a localized superior and inferior defect starting from the blind spot, while in the left eye, there was a superior nasal step with an initial arcuate defect, and an inferior nasal step with a localized defect.

An 84-year-old man with primary open-angle glaucoma (POAG) developed decompensated IOP in the right eye. Since 2003, his topical regimen has included tafluprost (15 ug/mL once daily), brinzolamide, and timolol (in combination, twice daily) and, during the last year, oral acetazolamide (250 mg twice daily) was added to the topical treatment. The preoperative best-corrected visual acuity (BCVA) was 6/10 in the right eye and 4/10 in the left eye (Snellen chart). The slit-lamp examination of the anterior segment revealed transparent cornea, normal anterior chamber, and nuclear lens opacity in both eyes with no other notable findings. The preoperative IOP was 24 and 20 mmHg in the right and the left eyes, respectively. On fundus examination, both eyes showed a large cup-to-disc ratio (CDR = 0.9) and neuroretinal rim narrowing in all the disc sectors. Despite the maximally tolerated medical therapy (both oral and topical), a noticeable bilateral visual field progression, most evident in the right eye, was confirmed in June 2020 (Figure 1). 

**Figure 2:**
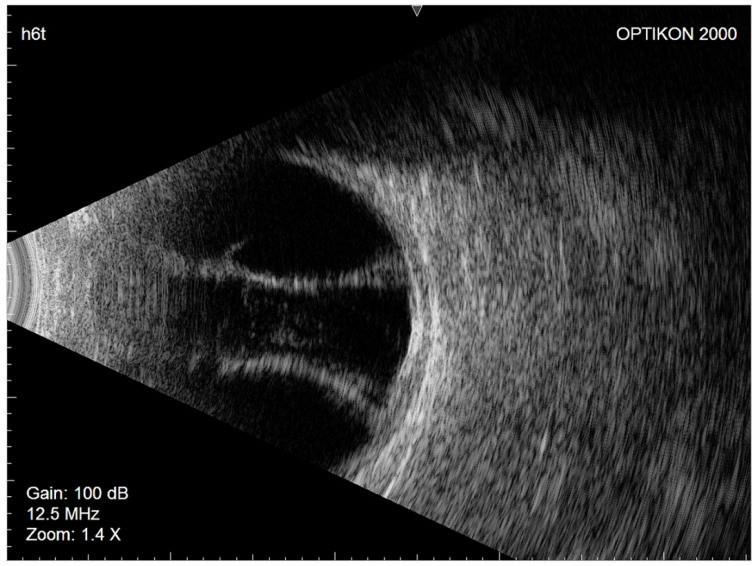
Right eye Eco-B scan showing serous choroidal detachment.

Considering the patient’s demographic and ocular characteristics and ideal IOP target, XEN45 implantation was selected as the most suitable glaucoma filtering surgery. In July 2020, XEN45 implantation with a subconjunctival injection of mitomycin C (0.2 mg/mL) was performed in the right eye. No intraoperative complications occurred. The day after surgery, the IOP was 10 mmHg, the filtration bleb appearance was excessively diffuse, and the anterior chamber was flat. Nasal and temporal CDs were visible on fundus examination, and their serous nature was confirmed by eco-B scan (Figure 2).

To counter possible inflammation and complications due to surgery and hypotony, topical dexamethasone (2 mg/mL, three times a day), atropine 1% (once a day), and oral prednisone (50 mg) were added to the routine postoperative antibiotic–corticosteroid combination. The patient was examined every week and showed a gradual improvement; after one month, the prednisone was gradually tapered, the IOP was 13 mmHg, and the CD completely resolved. 

**Figure 3:**
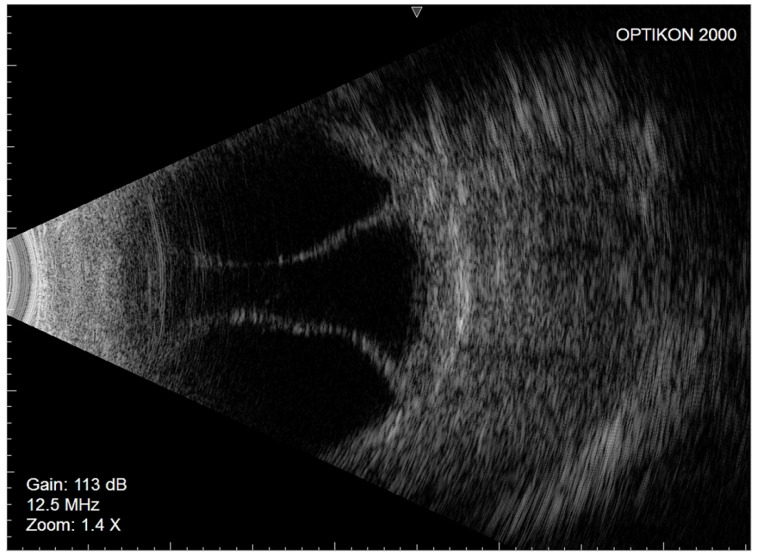
Left eye Eco-B scan showing serous choroidal detachment.

In March 2021, the left eye (similar to the fellow eye) underwent an uneventful ab interno XEN45 device implantation. The day after surgery, the IOP was 7 mmHg, the bleb morphology was diffuse, and the anterior chamber was deep. Seven days later, similar to the right eye, nasal and temporal CDs were observed on fundus examination and confirmed as serous CDs on eco-B scan (Figure 3).

The same topical and systemic therapies based on corticosteroids and cycloplegics were prescribed. However, due to persistent left CDs, surgical transscleral drainage was performed 2 months later to restore normal anatomy and avoid vision loss. After surgery, the IOP of the left eye stabilized between 10 and 12 mmHg, and the CD resolved permanently. On the slit lamp, the bleb appeared diffuse and normally vascularized, the anterior chamber was deep, and the gonioscopy evaluation showed a well-positioned XEN45 device in both eyes. BCVA in the left and right eye was affected by a dense subcapsular and nuclear lens opacity, so the best visual acuity could not be accurately assessed. 

In April 2022, phacoemulsification and intraocular lens (IOL) implantation were performed in the right eye after the risks and benefits of further surgery were discussed with the patient. During surgery, immediately after the capsulotomy, the lens material was aspirated through the hole with a small (30 gauge) needle to avoid the rupture of the capsulae due to swelling of the lens material. Then, capsulodesis and hydrodissection were performed, and phacoemulsification was completed. One week later, the BCVA in the right eye was 10/10 (Snellen chart), and the IOP was 10 mmHg. With the addition of the spherical lens + 3.00, the patient could read the 1° De Wecker (DW). Amsler’s test was slightly positive because of the pre-existing degeneration of the retinal pigment epithelium, but hypotony maculopathy apparently did not affect vision clinically. 

The phacoemulsification and IOL implantation were performed in the left eye in November 2022 using the same cataract surgical approach. One week after surgery, the BCVA was 6/10 (Snellen chart) and with the spherical lens + 3.00, the patient could read the 2° DW. Amsler’s test was positive because of the perifoveal accumulation of lipofuscin, classifiable as age-related macular degeneration (AMD), which was already observed in 2018.

## 3. Discussion

Different types of surgery are now available based on the anatomy of the eye, but hypotonia and CD are common complications for all glaucoma surgeries. The application of the XEN45 gel stent has been shown to reduce the risk of CD, making it a transient complication that generally does not have a negative effect on surgical outcomes. The use of an air bubble or viscoelastic material can help clinicians combat hypotonia by reforming the anterior chamber, which can appear from very shallow to completely flat with contact between the corneal endothelium and the iris. Both air and viscoelastic can close the tube and increase IOP, without the risk of them passing through the hole under the conjunctiva. In some cases, a peri-tube flow is observed, but this occurs only in the first few days and resolves spontaneously. This side effect could be reduced by the new XEN63 surgical device, which uses the same needle as the XEN45 to introduce the gel stent from the anterior chamber to the subconjunctival space, but this leaves a less empty space. In fact, compared to XEN45, the larger XEN63 stent could cause a more significant IOP reduction immediately after surgery, but this could guarantee a better result in the long follow-up.

According to recent studies, three main risk factors predispose patients to CD after XEN45 implantation: (i) older age, (ii) excess preoperative medications to lower IOP, and (iii) low IOP within one week after surgery [14]. Importantly, these risk factors were present in the clinical case reported here, in which persistent serous CD with visual loss occurred in both eyes after XEN45 implantation.

The XEN45 gel stent is commonly used to simplify surgery and limit postoperative complications [15]. Although the flow through the tube is not adjustable, the implant allows for a faster visual recovery and shorter surgical time than trabeculectomy. However, a careful preoperative evaluation is necessary to identify prognostic factors and, therefore, to select the most suitable patients for this type of surgery and to allow the patient to be adequately informed about any risk of complications.

As already mentioned, this type of filtering surgery with conjunctival bleb is faster than traditional trabeculectomy. In fact, it can be performed in 10–15 min with topical anesthesia, while trabeculectomy takes 30–45 min and requires peribulbar anesthesia. However, the brevity of the procedure and the use of topical anesthesia do not exclude the possible complications associated with this new type of surgery. Even though quick surgery avoids eye inflammation and topical anesthesia is preferred by older patients, CD can occur. Most cases of CD resolve spontaneously or with one to two weeks of treatment, but we have learned from clinical practice that although IOP should be kept low in the first few days after surgery, its lowering below 10 mmHg can have negative consequences. Indeed, as we recently reported, an IOP below 10 mmHg in the first 24–48 h after surgery should be considered a negative prognostic factor in the follow-up of XEN45 surgery [16]. 

In conclusion, the presented case highlights the importance of careful postoperative follow-up and timely intervention, and suggests that predisposing CD risk factors must be always taken into account in this surgery, even if less invasive and faster than trabeculectomy. In particular, special attention should be paid in case of CD in one eye, as it can represent a risk factor for CD in the other eye when undergoing the same type of surgery.

## Data Availability

The data presented in this study are available on request from the corresponding author.
